# Sessile serrated adenoma/polyps with a depressed surface: a rare form of sessile serrated adenoma/polyp

**DOI:** 10.1186/s13000-015-0325-x

**Published:** 2015-06-20

**Authors:** Eun-Jung Lee, Mi-Jung Kim, Sung-Min Chun, Se-Jin Jang, Do Sun Kim, Doo Han Lee, Eui Gon Youk

**Affiliations:** Department of Surgery, Daehang Hospital, Seoul, Republic of Korea; Department of Pathology, Daehang Hospital, 481-10 BangBae3-dong, Seocho-gu, 137-820 Seoul Republic of Korea; Department of Pathology, University of Ulsan College of Medicine, Asan Medical Center, Poongnap 2-Dong, Songpa-Gu, Seoul 138-736 Republic of Korea

**Keywords:** Sessile serrated adenoma, Colon, Endoscopy, Serrated polyp

## Abstract

Sessile serrated adenoma/polyps (SSA/Ps) usually appear flat to sessile with a smooth-appearing surface. However, macroscopic appearances of SSA/P can vary from flat-elevated to nodular and can even show a pedunculated configuration as we previously reported. The aim of the current study was to evaluate the clinicopathologic features of another under-recognized form of SSA/P which shows a depressed surface. Among 634 cases of sessile serrated adenoma/polyp, a total of seven sessile serrated adenoma/polyps showing a depressed surface were identified in 6 patients during the review of endoscopic images between January 2013 and November 2013. One of these was found during the review of previous endoscopic images of the same patient. Patients were more often middle-aged to elderly men (83.3 %) and had synchronous conventional adenomas and/or SSA/Ps except for one man. The polyps usually occurred in the proximal colon (71.4 %) and the mean size of polyps was 9.3 mm (range; 6-13 mm). Most cases (71.4 %) were of a flat-elevated type, and the remaining polyps (28.6 %) were sessile. The majority of polyps (85.7 %) showed a mucus cap. All but one of the cases (85.7 %) showed BRAF-V600E mutations. Our findings are that SSA/Ps can show a central depression although such cases are rare. The endoscopic and clinicopathologic features of SSA/Ps showing a depressed surface appear to be similar to usual SSA/Ps except for the presence of a depressed surface and marked male preponderance.

**Virtual slides:** The virtual slide(s) for this article can be found here: http://www.diagnosticpathology.diagnomx.eu/vs/1562070886167874.

## Background

With the understanding of the biologic nature of sessile serrated adenoma/polyps (SSA/Ps), its endoscopic detection has become more important [1, 2]. SSA/Ps are usually sessile to flat and similar in color to the surrounding mucosa. The presence of a mucus cap, a rim of bubbles or debris, alteration of the contour of a mucosal fold, and/or loss of the normal mucosal vascular pattern also suggests a possibility of an SSA/P [3]. Recently, the type II-O pit pattern has been introduced as a hallmark of an SSA/P [4]. However, some kinds of SSA/Ps are still under-recognized and poorly characterized despite increasing recognition of SSA/Ps among both endoscopists and pathologists. In a previous study, we described a pedunculated serrated polyp with histologic features of an SSA/P that could represent a pedunculated form of SSA/P in the spectrum of serrated neoplasia [5].

Herein we present seven cases of a previously undescribed form of SSA/P that shows a depressed surface. To the best of our knowledge, SSA/P with a depressed surface has not been reported in the English literature. We are reporting this rare form of SSA/Ps with the results of clinicopathologic and molecular analysis.

## Case presentation

**Fig. 1:**
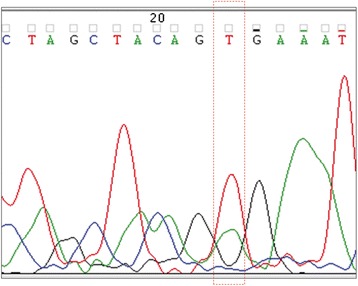
Representative example of sequencing result for BRAF. There was a GTG-to-GAG change (V600E) at codon 600 of exon 15

Table 1 lists the demographic and clinical information for the cases. All but one case were obtained from men. The mean age of seven cases was 57.3 years (range, 40-66). Most (71.4 %) polyps were removed from the proximal colon except for two cases from the sigmoid-descending junction. The polyps were relatively small, with a mean size of 9.3 mm (range, 6-13). Five of them were accompanied by synchronous tubular adenomas and two cases were associated with synchronous SSA/Ps. BRAF mutations were observed in all but one case of SSA/P showing a central depression. All mutations were a single missense mutation in codon 600 of exon 15 (V600E) (Fig. 1).

**Table 1:** Clinicopathologic and molecular features of SSA/Ps showing a depressed surface

Case	Age (years)	Sex	Location	Size (mm)	Endoscopic findings^a^	Procedure	Previous biopsy	BRAF mutation
1	40	M	Ascending colon	10	0-IIa + IIc, covered with mucus	EMR	-	V600E
2	59	M	Transverse colon	10	0-IIa + IIc, covered with mucus	Polypectomy	-	V600E
3	50	M	SD junction	8	0-Is + IIc	EMR	+	V600E
4	65	M	SD junction	13	0-Is + IIc, covered with mucus	EMR	+	V600E
5	66	F	Ascending colon	11	0-IIa + IIc, covered with mucus	EMR	+	V600E
6	62	M	Transverse colon	6	0-IIa + IIc, covered with mucus	Polypectomy	-	Negative
7	59	M	Splenic flexure	7	0-IIa + IIc, covered with mucus	EMR	-	V600E

F, female; M, male; SD, sigmoid-descending; EMR, endoscopic mucosal resection

^a^Paris classification [7]

## Materials and methods

A total of seven SSA/Ps showing a depressed surface were identified in six patients among 634 cases of SSA/P during the review of endoscopic images between January 2013 and November 2013. Among them, one case (case 7) was found during the review of previous endoscopic images of case 6. Patients were assessed for clinicopathologic features, including age and gender, anatomic site and size of polyp, and the presence and type of synchronous or metachronous polyps upon follow-up by reviewing the patients’ medical records, endoscopy and pathology reports. Permission for the study was obtained from the institutional review board at Daehang Hospital.

### Histology and mutation study

All specimens were routinely processed, stained with hematoxylin and eosin, and evaluated. The polyps included in this study were reviewed by M.J.K., who specializes in gastrointestinal pathology. SSA/Ps were defined by the abnormal architecture of the crypt bases, a so called “abnormal proliferation”, such as the branching of crypts, dilation of the base of the crypts, and inverted T- or L-shaped crypts [1, 2].

Genomic DNA was extracted from formalin-fixed, paraffin-embedded specimens. After DNA extraction, PCR amplification was performed as previously described [5, 6]. The amplified DNA products were purified and then direct sequencing was carried out using an ABI PRISM 310 sequence analyzer (Applied Biosystems, Foster City, CA).

### Colonoscopy

Colonoscopy was performed after standard bowel preparation. Total colonoscopies were carried out using a single-channel HD colonoscope (Olympus CF-H260AI; Olympus Optical, Tokyo, Japan). When a lesion was detected by colonoscopic examination, the surface mucus was washed away, and indigo carmine dye was spread over the lesion. The endoscopist (E.J.L.) retrospectively reviewed endoscopy images of the histologically proven SSA/Ps diagnosed between January 2013 and November 2013, reevaluating the morphology of polyps for size, anatomic location, gross appearance, and the presence of a mucous cap. The macroscopic morphology of polyps was characterized according to the Paris endoscopic classification (protruding sessile (Is) and protruding pedunculated (Ip); non-protruding superficial elevated (IIa), flat (IIb), and depressed (IIc) [7]. In this study, all the non-protruding neoplasms (IIa, IIb, IIc) were categorized as “flat”. The anatomic locations of polyps were grouped as proximal colon (cecum to splenic flexure) vs. distal colon and rectum combined.

## Results

The clinicopathologic and molecular features of the patients with SSA/Ps showing a depressed surface are summarized in Table 1. Patients were more often middle-aged to elderly men (83.3 %). Most (71.4 %) polyps were removed from the proximal colon except for two cases from the sigmoid-descending junction. The polyps tended to be relatively small, with a mean size of 9.3 mm (range; 6-13 mm). Five of them were accompanied by synchronous tubular adenomas and two cases were associated with synchronous SSA/Ps. BRAF mutations were observed in all but one case. All mutations were a single missense mutation in codon 600 of exon 15 (V600E) (Fig. 1).

**Fig. 2:**
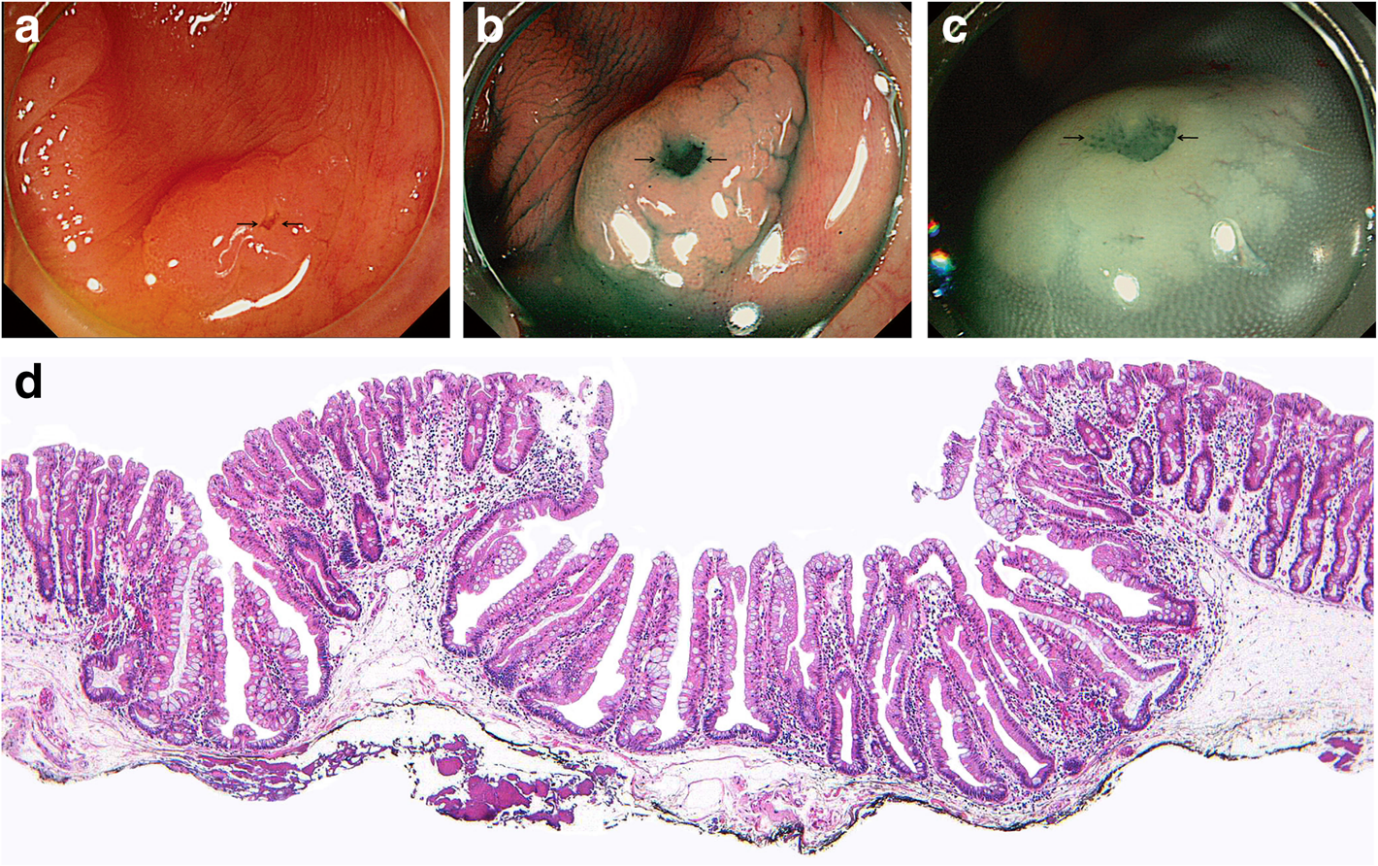
Endoscopic and histologic findings of case 1. **a**: Colonoscopy displayed a 10 mm laterally spreading tumor in the ascending colon. Most of the area of the polyp showed a flat-elevated surface with a mucous covering while the central portion displayed a depressed appearance (black arrows). **b** and **c**: Endoscopic images after the spraying of indigo carmine dye revealed a type II pit pattern in both the elevated and depressed areas (black arrows). **d**: A microscopic image showed typical histologic features of an SSA/P, such as dilatation and branching of basal crypts except for a central depression (H&E staining, x40)

All polyps were slightly elevated to sessile. The surface of the polyps was relatively smooth with a depressed surface (Fig. 2a,b,c). Five polyps showed a single depressed focus in the central portion, while the remaining two cases displayed surface depression in more than one area. The color of the polyps was slightly pale or the same as the normal mucosa. All but one of the polyps were covered with a mucus cap. The polyps showed an irregular or vague margin. Five (71.4 %) cases were removed by endoscopic mucosal resection and two were obtained by polypectomy.

On histopathological examination, both the flat-elevated and depressed areas showed dilatation and branching of the basal crypts, satisfying the criteria of an SSA/P (Fig. 2d). Histologic features suggesting other polyp or dysplasia were not noted in any of the cases. Histologic evidence of cytologic dysplasia such as nuclear hyperchromasia and elongation, pseudostratification, apoptosis, increased mitotic activity, and loss of mucin was not noted in any case.

## Discussion

In the current study, we describe ‘SSA/Ps of the colon with a depressed surface’ which share most clinicopathologic features with usual SSA/Ps. Most of these polyps are right-sided, covered with a mucous cap, and show BRAF-V600E mutations. In contrast to the tendency of usual SSAPs in women, these polyps frequently occurred in men [8].

According to the Paris classification [7], SSA/Ps with a depressed surface correspond to a type 0-IIa + IIc or 0-Is + IIc. While depressed type colorectal lesions have been reported to have greater risk of developing high-grade dysplasia or submucosal invasive cancer than other types [7, 9], there was no evidence of dysplasia in any case of SSA/P with a depressed surface in this study. However, larger-scale studies would be needed to confirm the prevalence rate of dysplasia and biologic nature of these polyps.

Besides under-recognition, there is also a potential risk for misdiagnosis as these polyps can resemble inverted colonic diverticulum (ICD), and vice versa [10]. Before spraying indigo carmine dye on the mucosal surface, the surface of these polyps can show only minimal mucosal changes. Therefore, if there is no knowledge about this kind of polyp, endoscopists can mistake this lesion for an ICD because these polyps can appear as a shiny, smooth-surfaced, polypoid lesion with a central indentation [10]. Although both lesions are rare, endoscopists should keep in mind both of these entities, as different approaches should be used depending on the judgement during endoscopy. If one favors an SSA/P, a complete removal of the lesion should be considered. However, in the case of ICD, an inadvertent attempt to remove the ICD endoscopically can cause serious complications, such as perforation [10].

In the current study, three of seven cases underwent a previous biopsy before EMR or polypectomy. Compared to the original shape, the central depression of the polyps was less noticeable in post-biopsy endoscopic images and polypectomy specimens. We suppose that a post-biopsy healing process results in biopsy scar and resultingly an architectural change in polyps. Therefore, previous endoscopic images need to be reviewed not to miss an SSA/P with a depressed surface if the patient had undergone a previous biopsy.

Finally, we suppose that the terminology “SSA/P” still has some drawbacks because the macroscopic appearance of SSA/Ps is not always sessile. Therefore, a new terminology reflecting their genuine nature might be necessary to reduce confusion in a clinical setting.

## Conclusion

We report the clinicopathologic and molecular features of a previously undescribed form of SSA/P. This type of polyp represents less than 1 % of all serrated polyps in our study. Clinically, SSA/Ps showing a depressed surface tend to develop in middle-aged to elderly patients, predilect for men, often occur in patients with conventional adenomas, usually arise in the proximal colon, and show BRAF-V600E mutations. The clinicopathologic features of SSA/Ps with a depressed surface seem to be similar to usual SSA/Ps except for the marked male preponderance and a depressed surface. However, a large-scale study would be necessary to confirm our observations because there is little data on this point.

## Consent

Written informed consents were obtained from the reviewed patients for publication of this case report and accompanying images. A copy of written consent is available for review by the Editor-in Chief of this Journal.

## Abbreviations

EMR: Endoscopic mucosal resection
ICD: Inverted colonic diverticulum
SSA/P: Sessile serrated adenoma/polyp
